# Using CRISPR/Cas9 genome editing in tomato to create a gibberellin‐responsive dominant dwarf DELLA allele

**DOI:** 10.1111/pbi.12952

**Published:** 2018-06-22

**Authors:** Laurence Tomlinson, Ying Yang, Ryan Emenecker, Matthew Smoker, Jodie Taylor, Sara Perkins, Justine Smith, Dan MacLean, Neil E. Olszewski, Jonathan D. G. Jones

**Affiliations:** ^1^ The Sainsbury Laboratory Norwich Research Park Norwich UK; ^2^ Department of Plant and Microbial Biology University of Minnesota St. Paul MN USA

**Keywords:** CRISPR/Cas9, DELLA, gibberellic acid, dwarf, slender, PROCERA

## Abstract

The tomato *PROCERA* gene encodes a DELLA protein, and loss‐of‐function mutations derepress growth. We used CRISPR/Cas9 and a single guide RNAs (sgRNA) to target mutations to the *PROCERA* DELLA domain, and recovered several loss‐of‐function mutations and a dominant dwarf mutation that carries a deletion of one amino acid in the DELLA domain. This is the first report of a dominant dwarf *PROCERA* allele. This allele retains partial responsiveness to exogenously applied gibberellin. Heterozygotes show an intermediate phenotype at the seedling stage, but adult heterozygotes are as dwarfed as homozygotes.

## Introduction

Genome editing methods have great promise for functional genomics research and crop improvement. The CRISPR (Clustered Regularly Interspaced Short Palindromic repeat)/Cas9 system is a powerful tool for genome editing. When this prokaryotic system is engineered for use in eukaryotes, a short RNA molecule, often expressed from an RNA polymerase III‐dependent promoter such as that of the U6 splicing factor RNA, guides the associated endonuclease Cas9 to generate double strand breaks 3 base pairs (bp) 5′ to the protospacer adjacent motif (PAM 5′‐NGG‐3′) in the target genomic DNA (Jinek *et al*., 2013; Ran *et al*., 2013; Sinkunas *et al*., 2013). Inaccurate repair of this break by non‐homologous end joining (NHEJ) within an exon often produces insertion/deletion (Indel) mutations that cause translational frame shifts or amino acid replacements or deletions.

The first reports of CRISPR/Cas9 editing in plants appeared in 2013. CRISPR/Cas9 editing has been reported in the model plants *Arabidopsis thaliana* and *Nicotiana benthamiana*, in rice, sorghum, wheat, tomato, grapes, maize and the opium poppy (Alagoz *et al*., 2016; Liu *et al*., 2017). CRISPR/Cas9 has now become the tool of choice for gene editing in plants not only to knock out gene(s) but also to insert or delete a gene (Filler Hayut *et al*., 2017; Roux *et al*., 2006) by using CRISPR/Cas9‐mediated DNA breaks to elevate levels of Homologous Recombination (Cermak *et al*., 2017; Cermák *et al*., 2015).

Plant growth is promoted by gibberellin, a hormone that is involved in diverse developmental processes, including stem elongation, leaf expansion, pollen development, flowering and seed germination. Gibberellins are diterpenoid acids that are synthesized by the terpenoid pathway in plastids and then modified further in the endoplasmic reticulum and cytosol to create the active form. The gibberellins are named GA_1_ through GA_*n*_ in order of discovery. GA3 was the first gibberellin to be identified, and although not the major active GA in most plants, was used in our experiments (Eriksson, 2006; Olszewski *et al*., 2002). Altering GA signalling disrupts control of cell and organ size (Fleet and Sun, 2005). GA promotes degradation of DELLA proteins, which are nuclear‐localized, negative growth regulators (Itoh *et al*., 2002; Olszewski *et al*., 2002; Wen and Chang, 2002). GA binding to the soluble Gibberellin Insensitive Dwarf (GID) receptor triggers GID interaction with the DELLA proteins, which then stimulates the assembly of an SCF E3 ubiquitin ligase complex that contains the GID2/SLEEPY1 F‐box proteins (Ueguchi‐Tanaka *et al*., 2005). This SCF complex polyubiquitinates the DELLA proteins, leading to their degradation by the 26S proteasome, thus derepressing growth (Livne *et al*., 2015). The *A. thaliana* genome encodes five highly homologous DELLA proteins including GA‐insensitive (GAI) and repressor of *ga1‐3* (RGA) (Peng *et al*., 1997). A 17‐amino acid deletion affecting the conserved DELLA motif results in a dominant dwarfing allele of GAI. Other dominant dwarfing alleles of the DELLA repressors include reduced height1 (Rht1) from wheat (*Triticum aestivum*), dwarf8 (d8) from maize (*Zea mays*) (Willige *et al*., 2007) and Sln1D from Barley (Chandler *et al*., 2002; Gubler *et al*., 2002). These alleles encode DELLA proteins that retain the ability to repress growth but are not destabilized by GA due to impairment in binding to the GA–GID1 complex. In most cases these dwarfs are non‐responsive to exogenous GA. Recessive loss‐of‐function DELLA (slender) mutants in barley, tomato and rice are highly elongated and male sterile (Asano *et al*., 2009; Chandler and Harding, 2013; Chandler *et al*., 2002; Ikeda *et al*., 2001). Arabidopsis and barley DELLA alleles have been found to uncouple the meristem and inflorescence size from plant height (Serrano‐Mislata *et al*., 2017).

Tomato is an important crop with 223 million tonnes produced in 2014 (Food and Agriculture Organization of the United Nations Database, 2014). Significant resources are invested for breeding traits such as disease resistance, fruit shape and colour. Tomatoes with reduced plant height and compact growth habits could be useful in some environments. Most currently grown glasshouse cultivars tend to be tall, trailing plants that require additional mechanical support, pruning and side‐shoot management (Hochmuth, 2001; Hochmuth *et al*., 1997). This study reports for the first time a dominant dwarf mutation in tomato using CRISPR technology, which may prove useful in the tomato industry.

## Results and discussion

### Editing the tomato DELLA‐encoding *PROCERA* gene

**Figure 1:**
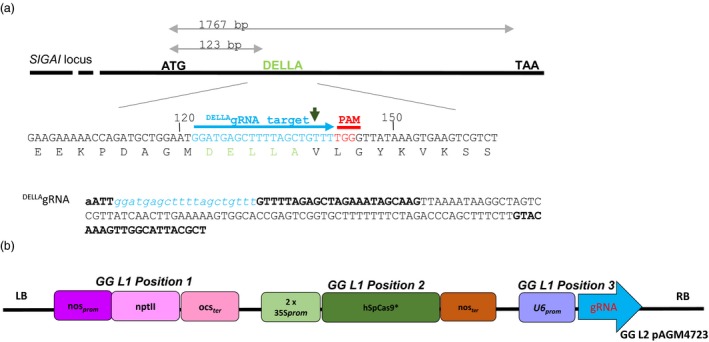
Design of gRNA targeting PROCERA. (a) Map of *PROCERA* locus. A single guide RNA (sgRNA) targeting the DELLA motif was designed as described in Nekrasov *et al*. (2013). The gRNA was designed to target the DNA sequence encoding the amino acid sequence DELLAVLG. The nuclease cleavage site is represented by the green arrow. (b). Map of the T‐DNA regions of the binary vector used to transform *Solanum lycopersicum* var. Moneymaker GCR758.

**Figure 2:**
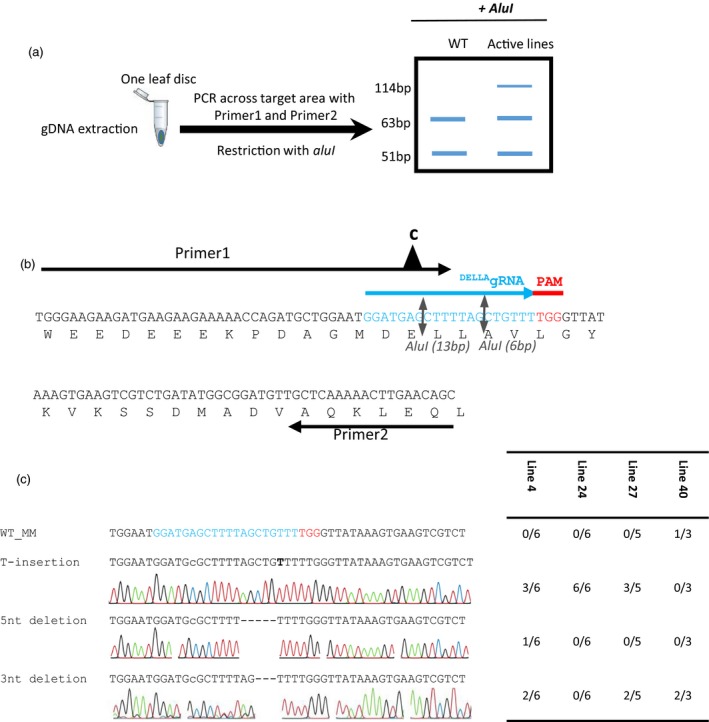
Restriction site loss assay used to test T_0_ plants. To detect mutations introduced by Cas9 nuclease in T_0_ plants, we assessed the loss of an AluI site due to imprecise NHEJ repair (Belhaj *et al*., 2013; Nekrasov *et al*., 2013; Voytas, 2013). (a) AluI cuts three nucleotides 5′ of where Cas9 is predicted to cut. In the fragment we chose to amplify, a second AluI recognition site is present 13 bp away from the PAM. PCR primers that removed the AluI site in the forward primer were designed to leave intact the second AluI site closer to the PAM, where it is likely to be altered by imperfect NHEJ. Two primers flanking the DELLA target locus were designed to amplify a fragment of 114 bp, digested with AluI and then visualized on a 3% agarose gel (51 and 63 bp). (b) The presence of AluI‐resistant bands in these plants indicates Cas9 nuclease activity in somatic tissues. Purified PCR fragments from line 4, 24, 27 and 40 were cloned into TA cloning vector and sequenced. (c) The analysis of the sequences reveals 3 types of mutations, either 5 or 3 nt deletions, or a T insertion.

We generated constructs carrying a single guide RNA that targets the nucleotides encoding the amino acids DELLAVLG of the tomato DELLA gene (Figure 1a,b) (see Experimental procedures). The DELLA target sequence GGATGAGCTTTTAGCTGTTT was submitted to a Blast search against tomato database using http://www.rgenome.net/cas-offinder/, and no other target was found. To detect mutations introduced by the Cas9 nuclease in T_0_ plants, we assessed loss of a restriction enzyme site that might have arisen due to imprecise NHEJ repair (Belhaj *et al*., 2013, 2015; Nekrasov *et al*., 2013; Voytas, 2013) (Figure 2a–c). Genomic DNA (gDNA) was extracted from the 46 T_0_ lines (individual transformants) (see Experimental procedures). For each of the T_0_ plants we PCR‐amplified the DELLA‐encoding domain and then digested with AluI and examined products on a 3% agarose gel (Figure S1). The AluI site is located 3 bp away from the Cas9 nuclease cutting site and small indels that extend into the AluI site should result in loss of AluI digestibility. We did not observe primary amplicons smaller than 114 bp, suggesting large deletions were not found in our experiments. Eight lines 4, 5, 6, 15, 23, 24, 27 and 40 showed a 114 bp non‐digested band in addition to two fragments (51 and 63 bp) produced when AluI digests wild‐type DNA, suggesting that the Cas9 nuclease was active in somatic tissues. Purified non‐digested 114 bp PCR fragments from lines 4, 24, 27 and 40 were cloned into a TA cloning vector and sequenced. Analysis of the sequences revealed three types of mutations: either a 5 or a 3 nucleotides (nt) deletion, or a T insertion (Figure 2c). Of the 46 T_0_ lines, only line 40 showed a strong AluI undigested band. Sequencing of the non‐digested band reveals the presence of wild type (WT) and DELLV fragments (Figure 2c).

### A heritable dwarf phenotype in progeny of T_0_ plants

**Figure 3:**
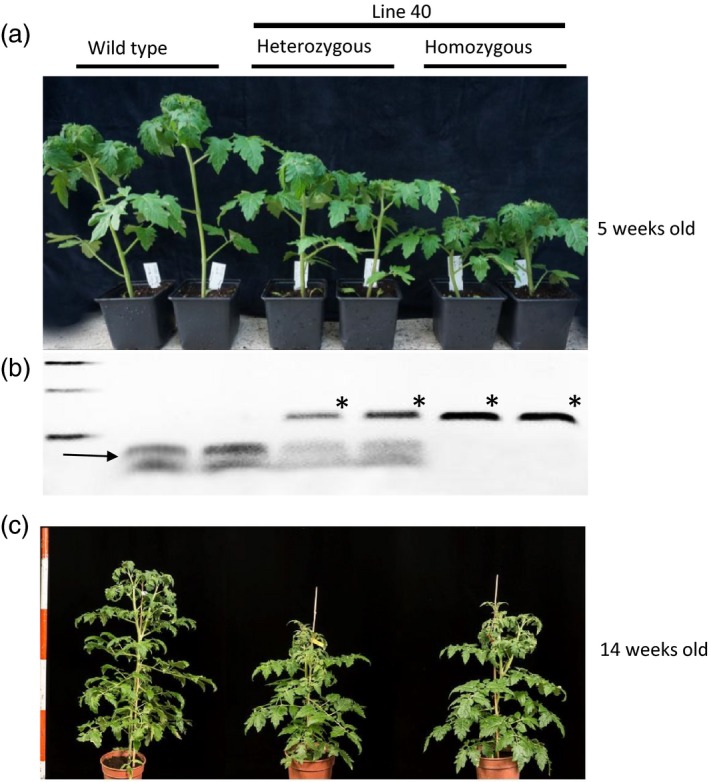
Thirteen T_1_ plants from line 40 were analysed phenotypically and by restriction enzyme site loss using AluI. (a) Phenotypic screening of self‐progeny from line 40 showed five plants that looked WT and eight plants that were smaller. (b) Genomic DNA from these individuals was extracted and PCR with primers flanking the target (Primer 1 and 2) was performed. Restriction enzyme site loss of these individuals reveal that three carried the AluI resistant band and therefore were homozygous mutants, five carried an AluI resistant band and two wild type bands so were heterozygous and five were wild type. The star indicates the DNA fragment resistant to AluI digestion. The arrow shows the 2 WT bands. (c) Early in development (5 weeks old) the heterozygous plants showed an intermediate phenotype between WT and homozygous, but later in development (14 weeks) heterozygous and homozygous plants for PRO^D^ looked the same.

T_0_ plants were self‐pollinated and T_1_ progeny plants were analysed either by restriction site loss assay or phenotypic screening. The fruits from lines 4, 5, 6 and 15 did not produce any seeds. When progeny from line 40 were grown, five of the plants had a WT stature and eight of the plants were smaller (Figure 3a) suggesting that an editing event might have occurred early during development (see also Figure 2c). Genomic DNA from these individuals was extracted and PCR with primers flanking the target (Primers 1 and 2) was performed. Restriction enzyme site loss analysis of these individuals revealed that three were homozygous mutants, five contained the AluI resistant band as well as the two WT bands and were scored as heterozygous, and five were WT (Figure 3b). The AluI‐resistant bands were cloned into T/A cloning vectors and sequenced. The comparison to the WT sequence revealed a 3 nt deletion at the predicted Cas9 editing site, leading to the conversion of the encoded amino acid sequence from DELLAVLG to DELLVLG. We named this mutation the *PRO* ^*D*^ allele of *PROCERA*. Both *PRO* ^*D*^/*PRO* ^*D*^ and *PRO* ^*D*^/*PRO* plants were smaller than WT (*PRO*/*PRO*) plants. Early in development, *PRO* ^*D*^/*PRO* plants showed an intermediate phenotype between WT and *PRO* ^*D*^/*PRO* ^*D*^ plants, suggesting that the mutation is semi‐dominant at the seedling stage. Later in development, *PRO* ^*D*^/*PRO* and *PRO* ^*D*^/*PRO* ^*D*^ plants are equally dwarfed compared to WT (Figure 3c). In Arabidopsis, GA perception results in DELLA protein ubiquitination by SCF^SLY/GID2^ and then degradation by the proteasome, resulting in DELLA protein destruction that de‐represses growth (Qin *et al*., 2014). Deletion of 17 amino acids containing the DELLA motif is responsible for the gain‐of‐function properties of the *gai‐1* allele which is severely dwarfed (Ariizumi *et al*., 2008). Several DELLA domain mutations have been described that result in GA‐insensitive growth in different plant species including barley, Arabidopsis, wheat and rice. Dominant dwarf DELLA protein forms are refractory to degradation upon GA perception. *PRO* ^*D*^/*PRO* tomato plants have one copy of the WT allele, and one copy of the DELLA mutant form that is expected to be refractory to degradation. Failure to degrade the DELLVLG protein causes growth retardation. It was also reported that there is a correlation between the severity of the dwarf phenotype and the stabilization of the DELLA protein caused by the mutation (Willige *et al*., 2007). The *dwarf8* alleles *D8‐1* and *D8‐Mp1* from maize show the same severe phenotype despite having deletions of different lengths within the DELLA domain.

### Generation of T‐DNA free plants carrying *PRO* ^*D*^/*PRO* ^*D*^ mutation

**Figure 4:**
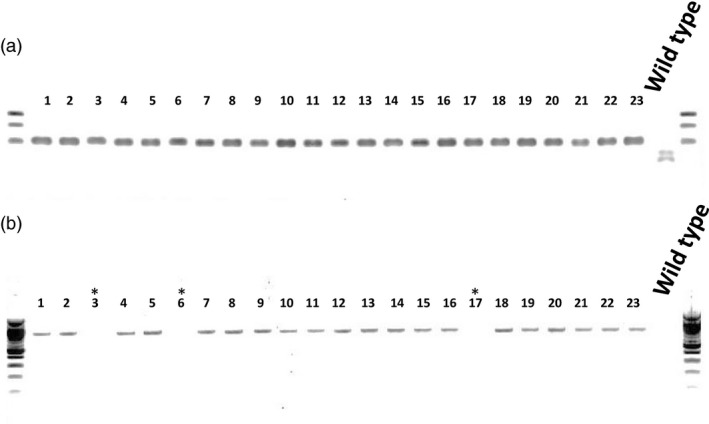
Cas9‐free plant detection. (a) Twenty‐three T_2_ progeny from line 40 were analysed for restriction site loss and the presence of the Cas9 gene. gDNA for each of the 23 individuals was extracted and then AluI restriction enzyme site loss after PCR amplification was tested. All the plants tested were PRO^D^/PRO^D^ homozygotes. (b) The same gDNA was subjected to PCR with the primers Cas9_fwd and Cas9_rev (see Experimental procedures) in order to detect the Cas9 transgene. The plants 3, 6 and 17 carry PRO^D^/PRO^D^ and no Cas9 transgene.

**Figure 5:**
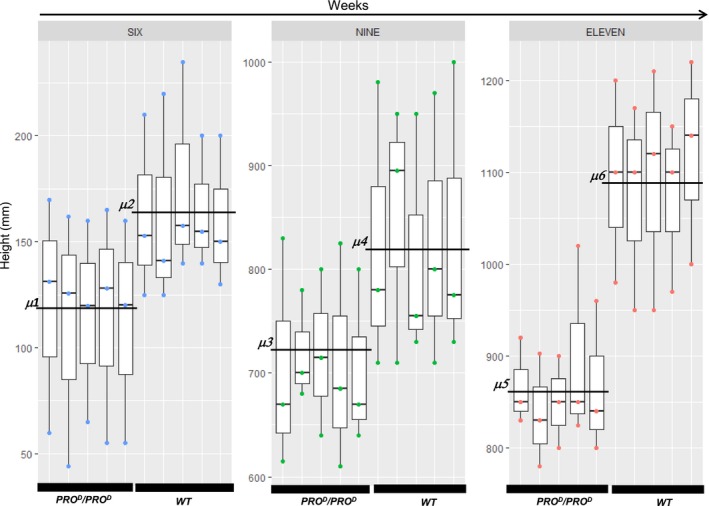
Size comparison between *PRO* ^*D*^/*PRO* ^*D*^ and WT at 6, 9 and 11 weeks after germination. Five plants (technical replicates) were monitored 6, 9 and 11 weeks after germination. Three biological replicates were conducted. For the 6 week time point, we observed the sample means μ2 = 165.44 ± 37 and μ1 = 114.72 ± 46.40. *P* < 0.00257 (ANOVA, Tukey). For the 9 week time point, we observed the sample means μ4 = 829.67 ± 113.16 and μ3 = 710.67 ± 76.71. *P* < 0.00219 (ANOVA, Tukey). For the 11 week time point, we observed the sample means μ6 = 1090.67 ± 96.69 and μ5 = 863.87 ± 65.32. *P* < 0.05 (ANOVA). These results show that *PRO* ^*D*^/*PRO* ^*D*^ plants are consistently smaller than the wild type (see Data S1).

To generate T‐DNA‐ free plants, *PRO* ^*D*^/*PRO* ^*D*^ dwarf mutant T_2_ plants from line 40 were screened by PCR not only for the absence of AluI digestion but also for the absence (due to genetic segregation) of the Cas9 transgene using primers that amplify 858 bp of Cas9. Of 23 plants tested, three plants carried the AluI resistant band and no Cas9 transgene (Figure 4). *PRO* ^*D*^/*PRO* ^*D*^ plants exhibit a phenotype resembling GA‐deficient mutants, with darker green leaves, shorter stems and internodes and late flowering, which is consistent with what has been reported in the literature for other ‘dominant’ DELLA dwarf mutants (Figure 5). *PRO* ^*D*^/*PRO* ^*D*^ mutants are smaller than WT plants at 6, 9 and 11 weeks old (Figure 5). The diameter of *PRO* ^*D*^/*PRO* ^*D*^ fruit was slightly but not significantly smaller than WT; however, fruit weight was slightly and significantly reduced (Data S1). The *PRO* ^*D*^/*PRO* ^*D*^ fruits contain an average of 35 fewer (~57 versus ~92) seeds per fruit compared to the WT (Figure S2). The flowering time and fruit setting time were indistinguishable between the two genotypes (Figure S3). The number of tomato fruit per plant was calculated and show that *PRO* ^*D*^/*PRO* ^*D*^ and WT produce the same average number of fruits per plant.

### *PRO* ^*D*^/*PRO* ^*D*^ plants retain partial responsiveness upon GA treament

**Figure 6:**
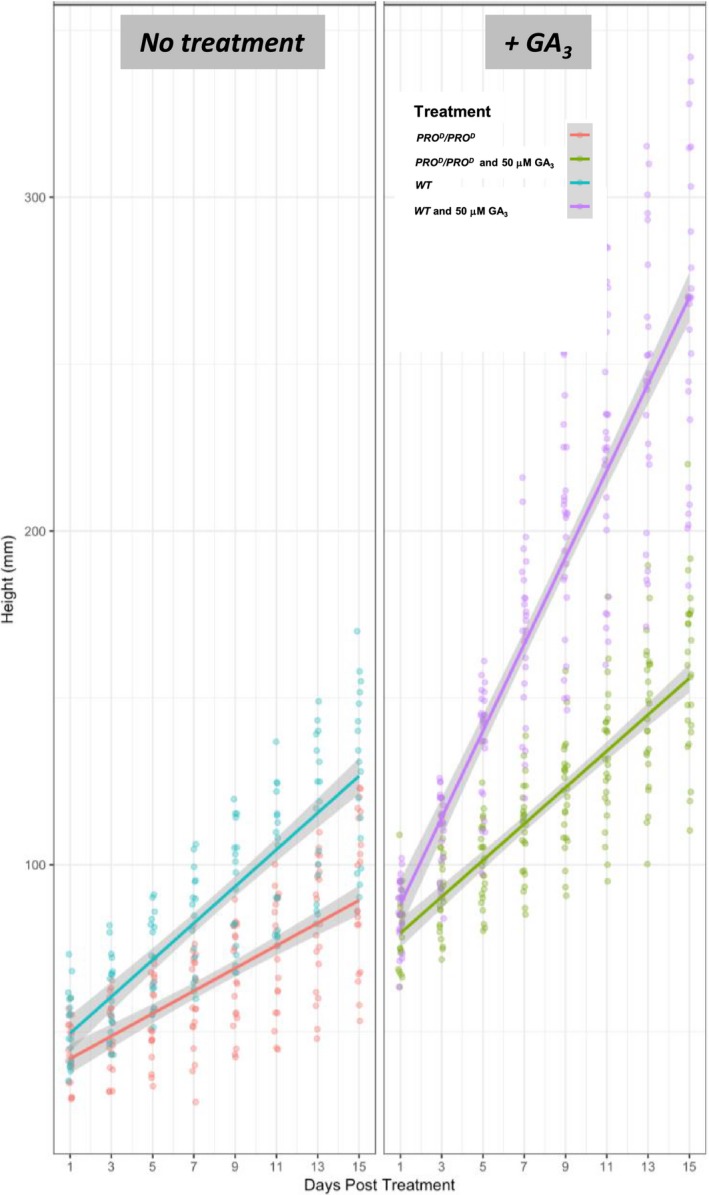
Growth comparison of homozygous *PRO* ^*D*^/*PRO* ^*D*^ and WT plants upon application of GA_3_ (50 μm). Scatter plot of height versus days post‐treatment with 50 μm GA_3_. Lines represent linear model regression ±SD (Data S1). Each spot corresponds to a biological replicate. These data show reduced but not abolished GA_3_ responsiveness in *PRO* ^*D*^/*PRO* ^*D*^ homozygotes.

We investigated the GA response of *PRO* ^*D*^/*PRO* ^*D*^ tomato lines. Mutant and WT plants were sprayed to run‐off with 50 μm gibberellic acid (GA_3_) (see Experimental procedures). The results showed that *PRO* ^*D*^/*PRO* ^*D*^ plants, while growing slower than WT, retain partial responsiveness to GA treatment (Figure 6). A similar observation was made on the barley Sln1D allele where a single amino acid exchange mutation DELLAVLG to DELLAVLE showed an intermediate phenotype with respect to plant growth and mutant GAI protein stabilization (Chandler *et al*., 2002; Gubler *et al*., 2002; Willige *et al*., 2007). A dwarf maize mutant was also reported carrying a single amino acid insertion in a domain located downstream of the DELLA domain, called VHYNP domain of the *dwarf8* gene and shows a strongly reduced but not abolished response to GA (Cassani *et al*., 2009). Further studies with the Arabidopsis GID1A GA receptor and the GAI DELLA repressor mutant proteins indicate that the loss (or reduction in the case of Sln1D) of GA responsiveness can be explained by the loss (or reduction) of GA‐dependent GID1A binding. Thus, our observations are consistent with other reports in the literature that partial interference with DELLA degradation by small deletions in the DELLA domain result in a weak or partially dominant dwarf phenotype (Chandler *et al*., 2002; Gubler *et al*., 2002; Willige *et al*., 2007).

### Recovery of *PROCERA* loss‐of‐function mutants in the progeny of T_0_ plants

**Figure 7:**
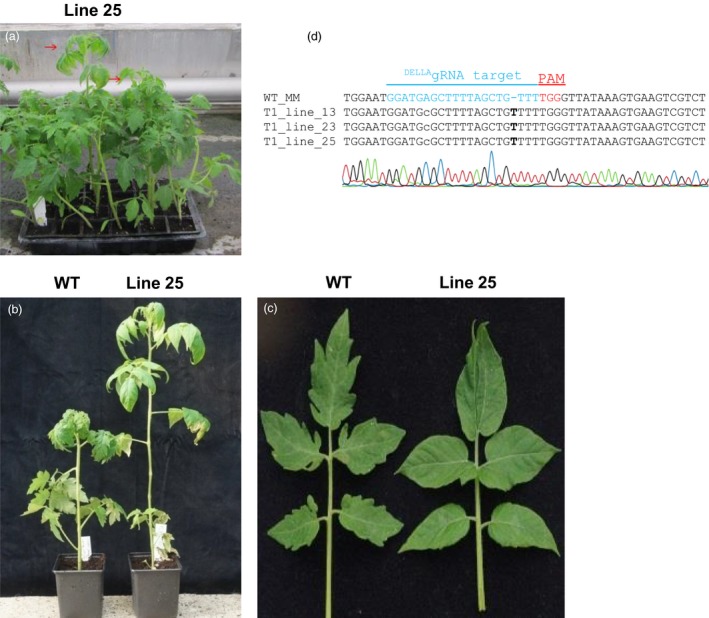
DELLA loss‐of‐function mutants show a *PROCERA‐like* phenotype. Progeny of lines 13, 23 and 25 include plants having a *PRO* mutant phenotype (red arrows) (a). Two plants from line 25 with increased stature compared to wild type (a, b) and smooth leaf margin (c) characteristic of *PROCERA* mutants. Genomic DNA from these individuals was extracted, PCR with primers flanking DELLA‐encoding domain was performed and products were subsequently cloned into T/A cloning vectors. Sanger sequencing of the ^DELLA^gRNA target region showed a T insertion at Cas9 nuclease cutting site leading to a frame shift after the DELLA motif (‐MDELLAVFGL*) (d). Alignment of CRISPR/Cas9 targets of three lines of transformed tomato plants showing *PRO*‐like mutant phenotype.

Several lines 1, 13, 23, 25, 33 and 46 that showed no alteration in AluI restriction pattern (2 bands of 51 and 63 bp) were sown alongside WT plants (Figure 7a). When five, 2 and 72 individuals from lines 13, 23 and 25 were grown, one, one and three plants respectively in these populations were taller than their siblings. Similar to what has been observed in *PROCERA* mutants (Bassel *et al*., 2008; Jasinski *et al*., 2008), the leaves of these individuals bear fewer leaflets and the leaflets have a smoother margin (Figure 7b,c). Genomic DNA from these individuals was extracted, PCR‐amplified with primers flanking the DELLA‐encoding domain (Primer 1 and 2) and PCR products cloned into T/A cloning vectors. Sequencing analysis revealed a T insertion next to the PAM motif leading to an early stop codon just after the DELLA motif for all of them (‐MDELLAVFGL*) (Figure 7d), such mutations would not be revealed by the AluI digestion assay since the AluI site is located 3 bp away from the Cas9 nuclease cutting site. The previously reported *PROCERA* mutant phenotype was due to a T905A mutation leading to the conversion of VHVID to VHEID in the C‐terminal GRAS motif, which is thought to be important for DELLA action but has no defined biochemical function. A *PRO* mutant allele was obtained using TALENs in order to determine the role of PROCERA in GA signalling (Livne *et al*., 2015). In contrast with the first characterized *PROCERA* allele which carries a missense mutation and shows partial responsiveness to GA, isolated proTALEN and pro∆GRAS mutations are complete null alleles and GA insensitive (Livne *et al*., 2015). The targeting of the DELLA motif using CRISPR Cas9 in our experiments thus resulted in the generation of two phenotypes, one (*PRO* ^*D*^/*PRO* ^*D*^ *)* showing a dwarf phenotype and the second a *PROCERA*‐like phenotype caused by loss of PROCERA function.

## Conclusion

CRISPR technology is a powerful tool for genome editing that enables modification of gene(s) of interest, and since the target DNA is unlikely to be linked to the T‐DNA, the Cas9 T‐DNA locus can be segregated away by crossing. We report here the first use of CRISPR technology to engineer a dominant gain‐of‐function mutation that potentially could confer a desirable commercial trait. Over the past decades, DELLA gene mutations have been used by plant breeders. The most notable example is the dominant semi‐dwarf wheat cultivars, a crucial component of the ‘green revolution’ genes which all contain deletions in the DELLA region of an Rht gene (Pearce *et al*., 2011; Peng *et al*., 1999). Our report paves the way for facile provision of dominant semi‐dwarf alleles in other crops.

## Experimental procedures

### Plant growth and transformation

Transformations were conducted using the tomato cultivar Moneymaker GCR758 (*Solanum lycopersicon*). The binary Level 2 vector pAGM4723 containing the cassette nos_*prom*_:nptII:ocs_*ter*_, 2x35S_*prom*_:hSpCas9:ocs_*ter*_ and ^DELLA^gRNA were mobilized into *Agrobacterium tumefaciens* Agl1. Transgenic plants were regenerated by standard methods for tomato transformation (Fillatti *et al*., 1987). All the independent transformed lines were analysed. Plants derived from tissue culture were rooted in Jiffy peat plugs. Plants were potted into 7 cm pots using Levington M2 peat based compost and placed in a closed unheated propagator in the glasshouse. After 24 h in the propagator, they were vented (approx. 5%) and the resilience of the plants to external glasshouse conditions was tested. Plants were gradually acclimatized to the glasshouse conditions and then grown in Levington's M3 compost (Levington Horticulture Ltd., Fisons, Ipswich, UK) in a greenhouse at temperatures between 22 and 27 °C in the light and 12–16 °C in the dark. Sixteen hours of light was supplied at a photon flux density of 300–650 μE/m^2^/s at the plant surface, and the relative humidity was between 70% and 80%.

### Molecular cloning

#### Golden Gate Plasmid constructs

We used the methods for designing single guide RNAs (sgRNAs) described in Nekrasov *et al*. (2013) to target the DELLA motif. The DELLAVLG amino acid sequence of PROCERA (PRO) is encoded by GATGAGCTTTTAGCTGTTTTGGGT, and we chose GGATGAGCTTTTAGCTGTTT as the target sequence with TGG as the PAM. This sequence was fused to a single gRNA (Jinek *et al*.,^2012^). The 20nt ^DELLA^gRNA was amplified with the following primers ^DELLA^gRNA_F gaGAAGACaaATT*ggatgagcttttagctgttt*GTTTTAGAGCTAGAAATAGC and ^DELLA^gRNA_R gaGAAGACaaAGCGTAATGCCAACTTTGTAC using as a template the Addgene Plasmid # 46966. The 158 bp resulting fragment was gel purified using a Qiagen gel extraction kit and subsequently cloned into the L0 module using Golden Gate technology (Engler *et al*., 2009, 2014; Patron, 2014). RNA Pol III transcribes U6 RNA genes of Arabidopsis, and a functional U6 promoter contains an Upstream Sequence Element and TATA‐like elements. We engineered a U6 synthetic promoter which is the consensus sequence of the three U6 promoter variants identified in the Arabidopsis genome (Nekrasov *et al*., 2013; Waibel and Filipowicz, 1990). The resulting Level 2 binary vector (L2) in pAGM4723 carries a kanamycin resistance cassette in position 1 (Addgene Plasmid # 51144), 2x35S_*prom*_:hSpCas9:ocs_*ter*_ in position 2 (Addgene plasmid # 49771) and U6:^DELLA^gRNA in position 3 (Figure 1b) (Engler *et al*., 2014; Patron, 2014). BsaI‐HF and BbsI from New England Biolabs and Thermo Fisher Scientific respectively were also used for the building up of the Golden Gate constructs.

#### T/A cloning and Sanger sequencing

We used the pGEM‐T kit from Promega, Southampton, United Kingdom for TA cloning of PCR products. The Sanger sequencing of the constructs was done by GATC Biotech.

#### Genomic DNA extraction, restriction loss assay and PCR assays

The majority of DNA extraction methods from plant leaf tissue are derived from the original hexadecyltrimethylammonium bromide (CTAB)‐based method described by (Doyle and Doyle, 1987). One hundred nanograms of DNA from each individual was used to perform a PCR with Primer1 GGGAAGAAGATGAAGAAGAAAAACCAGATGCTGGAATGGATG**C**GCTT and Primer2 GCTCAAAAACTTGAACAGC. Oligonucleotides were purchased from Merck, Gillingham, Dorset, Great Britain. PCR amplifications were conducted using New England Biolabs, Wilbury Way, Hitchin, Hertfordshire, Taq DNA polymerase (Catalogue: M0273S) and following the manufacturer protocol with a 25 μL final volume. Mixtures were amplified in a Bio‐Rad C1000 Touch PCR machine Watford, Hertfordshire, UK. The thermal cycle was programmed for 30 s at 95 °C as initial denaturation, followed by 35 cycles of 30 s at 95 °C for denaturation, 30 s at 55 °C as annealing, 15 s at 72 °C for extension, and final extension at 72 °C for 5 min. Five microliters of the PCR products were examined by electrophoresis at 100 V for 30 min in a 3% (w/v) agarose gel in 1× TBE buffer containing ethidium bromide. The gel was then observed under UV light. Restriction loss assay was conducted according to (Belhaj *et al*., 2013) using AluI. One unit per reaction of AluI from New England Biolabs was used for the restriction digest. PCRs to detect the presence of Cas9 gene were conducted in the same conditions as described above except the extension was 45 s and the PCR products were examined by electrophoresis in a 1.5% (w/v) agarose gel; the primers used were Cas9_Fwd: TTTCAGATTTCAGAAAGGACTTTCAG and Cas9_Rev: AGAtCCTTTGAGCTTTTCATAGTGGCTGG.

### Gibberellic acid growth assays

Tomato seeds were planted in BM2 Germinating Mix (Berger Peat Moss, Saint‐Modeste, Quebec, Canada) initially saturated with 1.5 g/L 20 : 10 : 20 20‐10‐20 Peat‐Lite Fertilizer (JR Peters Inc, Allentown, PA). Tomato plants were grown for 4 weeks in flats spaced such that they would not significantly impede each other's growth. After 4 weeks, the tomato plants were measured and then treated by spraying to runoff with 50 μm GA_3_ (Sigma, Gillingham, Dorset, United Kingdom; G7645; day 1 of treatment). As the GA_3_ stock was dissolved in ethanol, the control tomato plants were treated with water containing the equivalent amount of ethanol. The GA_3_ stock solution consisted of 50 mm GA_3_ in 70% ethanol, and 1 mL/L was added to make the final 50 μm solution. In the control solution, 1 mL/L 70% ethanol was added. Plants were treated on days 1, 3 and 5. Measurements were taken on days 1, 3, 5, 7, 9, 11, 13 and 15.

## Author contributions

J.D.G.J and L.T. wrote the manuscript, J.D.G.J. and LT designed the experiments which were conducted by LT and YY. R.E. and N.O. designed and did GA experiments. S.P. and J.S took care of plants and helped with phenotyping. D.M helped with statistical analysis of the results and M.S. and J.T. made transgenic plant lines. Andrew Davis (JIC photographic department) helped with photography.

## Conflict of interest

The authors declare no conflict of interest.

## Supporting information


**Figure S1** Restriction site loss assay of the 46 T_0_ tomato Moneymaker lines.

**Figure S2** Graphs showing the seed number per fruit, fruit diameter (mm), height (mm) and weight (gram) for PRO^D^/PRO^D^ and WT.

**Figure S3** Flowering time and flower setting into fruit of PRO^D^/PRO^D^ tomato mutant plants versus WT.

**Data S1** Raw data, R Markdown and html documents.
