# Supplementary material for: Using CRISPR/Cas9 genome editing in tomato to create a gibberellin‐responsive dominant dwarf DELLA allele
Source: Plant Biotechnol J. 2018 Jun 22;17(1):132–40. doi: 10.1111/pbi.12952 (PMC6330640; doi:10.1111/pbi.12952)
Supplement: Supplementary file 1 — Figure S1 Restriction site loss assay of the 46 T0 tomato Moneymaker lines. [file PBI-17-132-s003.pdf]

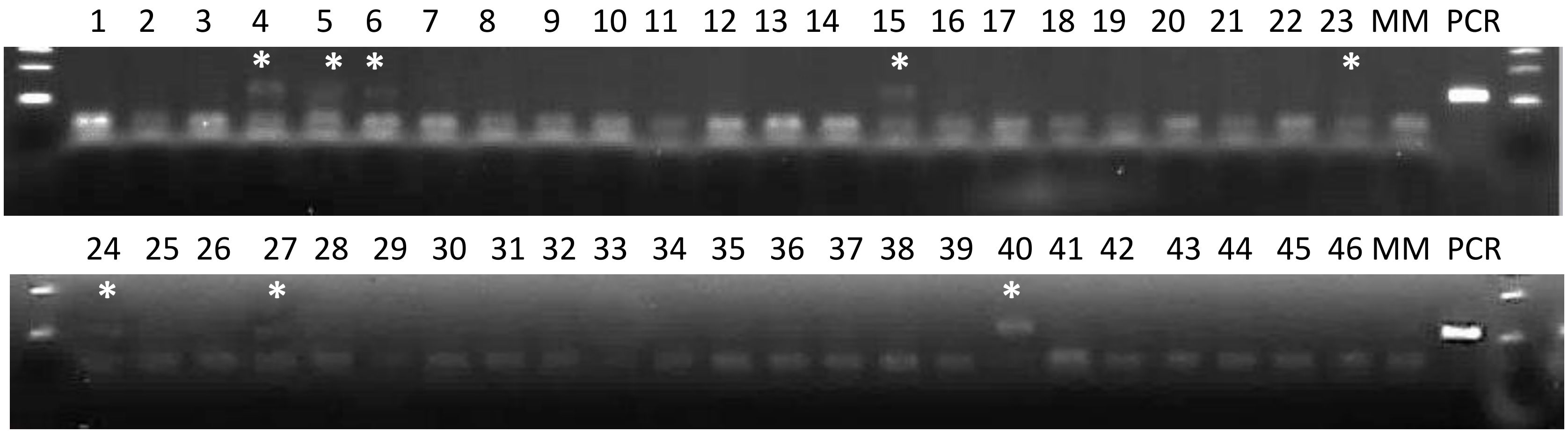

Supplemental Figure S1: Restriction site loss assay of the 46 T_0 tomato Moneymaker lines.
After PCR amplification, AluI digestion products were run on a 3% agarose gel. * indicates the lines with Cas9 nuclease activity. Line 4, 5, 6, 15, 23, 24, 27 and 40 showed a resistant band. Tomato var. Moneymaker WT and PCR product before AluI restriction.
