# Supplementary material for: Using CRISPR/Cas9 genome editing in tomato to create a gibberellin‐responsive dominant dwarf DELLA allele
Source: Plant Biotechnol J. 2018 Jun 22;17(1):132–40. doi: 10.1111/pbi.12952 (PMC6330640; doi:10.1111/pbi.12952)
Supplement: Supplementary file 2 — Figure S2 Graphs showing the seed number per fruit, fruit diameter (mm), height (mm) and weight (gram) for PROD/PROD and WT. [file PBI-17-132-s004.pdf]

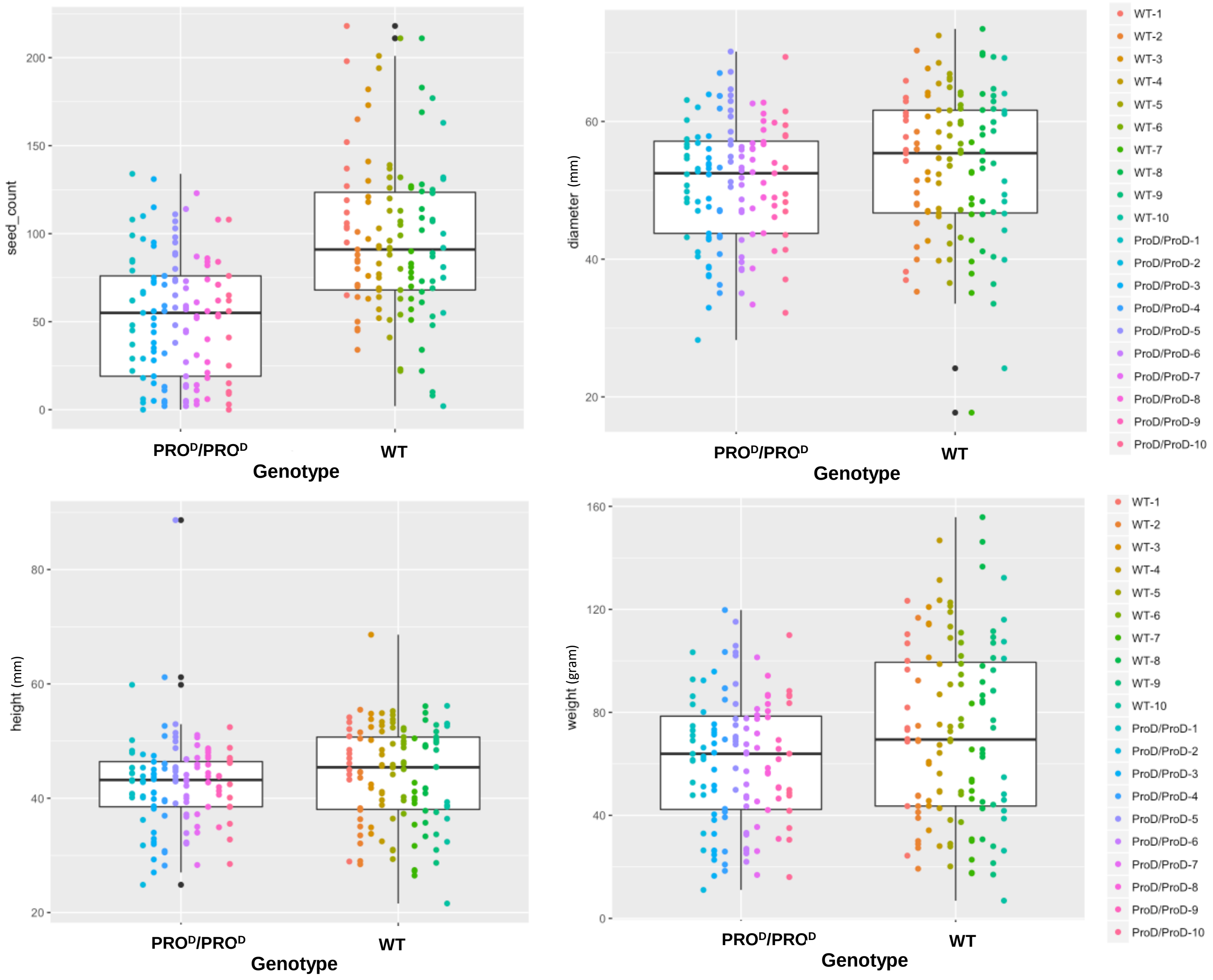

Supplemental Figure S2: **Graphs showing the seed number per fruit, fruit diameter (mm), height (mm) and weight (gram) for PRO^D/PRO^D and WT.**

Ten plants from each genotype were recorded. Each coloured dot represents a fruit. **a.** Seed count of fruits produced by PRO^D/PRO^D and WT. Linear regression analysis revealed that PRO^D/PRO^D fruit contain ~35 seeds fewer than WT (~57 versus ~92) with p< 2e-16. **b.** Diameter in mm of tomato fruit produced by either PRO^D/PRO^D or WT. Linear regression analysis shows no significant difference between the two. **c.** Fruit height in mm of PRO^D/PRO^D mutant and WT. Linear regression analysis shows no difference between the two. **d.** Weight in grams of the fruit for PRO^D/PRO^D mutant and WT. Linear regression analysis revealed that overall PRO^D/PRO^D fruit are ~0.9 grams lighter than the WT (~65g versus ~66g) with p< 1.59e-05. The code to generate these plots is represented as R Markdown and html in Supplemental Data S1.
