# Supplementary material for: Using CRISPR/Cas9 genome editing in tomato to create a gibberellin‐responsive dominant dwarf DELLA allele
Source: Plant Biotechnol J. 2018 Jun 22;17(1):132–40. doi: 10.1111/pbi.12952 (PMC6330640; doi:10.1111/pbi.12952)
Supplement: Supplementary file 3 — Figure S3 Flowering time and flower setting into fruit of PROD/PROD tomato mutant plants versus WT. [file PBI-17-132-s002.pdf]

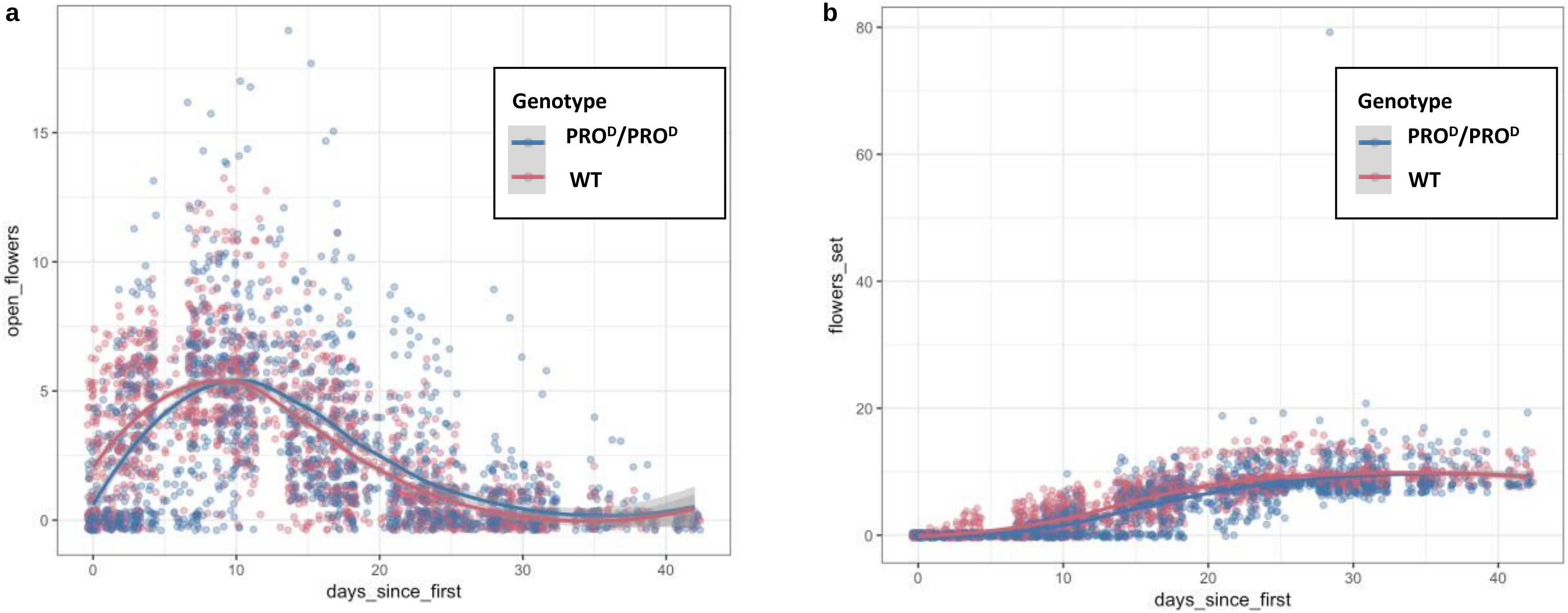

Supplemental Figure S3: Flowering time and flower setting into fruit of PRO^{D}/PRO^{D} tomato mutant plants *versus* WT.
a. Graph representing flower opening time of PRO^{D}/PRO^{D} mutants and WT. ANOVA analysis shows no difference between the two. **b.** Graph representing the numbers of flowers that set fruit. ANOVA analysis show no difference between the two. We can conclude that flowering time and flower setting into fruit is the same for PRO^{D}/PRO^{D} and WT plants. The code used to generate these plots is presented as R Markdown and html document in Supplemental data S1.
