# Supplementary material for: Using CRISPR/Cas9 genome editing in tomato to create a gibberellin‐responsive dominant dwarf DELLA allele
Source: Plant Biotechnol J. 2018 Jun 22;17(1):132–40. doi: 10.1111/pbi.12952 (PMC6330640; doi:10.1111/pbi.12952)
Supplement: Supplementary file 4 — Data S1 Raw data, R Markdown and html documents. [file PBI-17-132-s001.zip › Data S1/Supporting data for Fig 6/GA_assay_lm.html]

Untitled


# Untitled

## R Markdown

This is an R Markdown document. Markdown is a simple formatting syntax for authoring HTML, PDF, and MS Word documents. For more details on using R Markdown see http://rmarkdown.rstudio.com.

When you click the **Knit** button a document will be generated that includes both content as well as the output of any embedded R code chunks within the document. You can embed an R code chunk like this:

```
library(ggplot2)
# load data
GA_Assay <-read.csv('GA_Assay.csv')

#change rep to factor from integer
GA_Assay$Rep <- as.factor(GA_Assay$Rep)

#add genotype ga nteraction column
GA_Assay$geno_ga <- with(GA_Assay, interaction(Genotype, GA_50ul))
str(GA_Assay)
```

```
## 'data.frame':    704 obs. of  7 variables:
##  $ Genotype: Factor w/ 2 levels "DELLA","WT": 2 2 2 2 2 2 2 2 2 2 ...
##  $ DPT     : int  1 3 5 7 9 11 13 15 1 3 ...
##  $ GA_50ul : logi  FALSE FALSE FALSE FALSE FALSE FALSE ...
##  $ Height  : int  67 75 80 85 91 95 100 110 85 99 ...
##  $ Plant   : int  1 1 1 1 1 1 1 1 2 2 ...
##  $ Rep     : Factor w/ 3 levels "1","2","3": 1 1 1 1 1 1 1 1 1 1 ...
##  $ geno_ga : Factor w/ 4 levels "DELLA.FALSE",..: 2 2 2 2 2 2 2 2 2 2 ...
```

```
#basic plot
p <- ggplot(GA_Assay) + aes(x=DPT, y=Height, colour=factor(geno_ga, labels= c("DELLV", "DELLV and GA", "WT", "WT and GA"))) + geom_jitter(width=0.1, alpha=0.4) + geom_smooth(method = "lm") + facet_grid(. ~ Genotype)
#labels=c("DELLV", "DELLV and GA", "WT", "WT and GA")
```

```
p + labs(colour = "Treatment", x = "Days Post Treatment", y = "Height (mm)") + theme_bw() + scale_x_continuous(breaks = seq(1,15, by=2))
```

```
ggsave("final_plot.png",width = 12, height = 8)
```

```
save(GA_Assay, file="GA_Assay.rda")
```

```
lm.fit <- lm(Height ~ DPT + Genotype:GA_50ul, data = GA_Assay)
summary(lm.fit)
```

```
## 
## Call:
## lm(formula = Height ~ DPT + Genotype:GA_50ul, data = GA_Assay)
## 
## Residuals:
##     Min      1Q  Median      3Q     Max 
## -65.485 -19.317   1.273  16.676 112.341 
## 
## Coefficients: (1 not defined because of singularities)
##                             Estimate Std. Error t value Pr(>|t|)    
## (Intercept)                 121.2588     2.6374   45.98   <2e-16 ***
## DPT                           7.2267     0.2269   31.85   <2e-16 ***
## GenotypeDELLA:GA_50ulFALSE -113.4784     2.9014  -39.11   <2e-16 ***
## GenotypeWT:GA_50ulFALSE     -61.3821     2.7325  -22.46   <2e-16 ***
## GenotypeDELLA:GA_50ulTRUE   -91.0648     3.0427  -29.93   <2e-16 ***
## GenotypeWT:GA_50ulTRUE            NA         NA      NA       NA    
## ---
## Signif. codes:  0 '***' 0.001 '**' 0.01 '*' 0.05 '.' 0.1 ' ' 1
## 
## Residual standard error: 27.59 on 699 degrees of freedom
## Multiple R-squared:  0.7986, Adjusted R-squared:  0.7974 
## F-statistic: 692.8 on 4 and 699 DF,  p-value: < 2.2e-16
```
