# Supplementary material for: Using CRISPR/Cas9 genome editing in tomato to create a gibberellin‐responsive dominant dwarf DELLA allele
Source: Plant Biotechnol J. 2018 Jun 22;17(1):132–40. doi: 10.1111/pbi.12952 (PMC6330640; doi:10.1111/pbi.12952)
Supplement: Supplementary file 4 — Data S1 Raw data, R Markdown and html documents. [file PBI-17-132-s001.zip › Data S1/Supporting data for Fig S2/seed_effects.html]

GA Seed Effects


# GA Seed Effects

#### *Dan MacLean*

#### *04/12/2017*

```
library(tidyverse)
library(stringr)
```

## Load in the data

```
data <- read_csv(
  "Wt_vs_DELLA_seed.csv", 
  skip = 1,
  col_types = cols(
    gid = col_factor(NULL) 
  ),
  col_names = c("gid","seed_count", "diameter","height", "weight")
) %>%
  mutate(
    is_wt = str_detect( gid, "WT"),
    genotype = factor(if_else(is_wt, "WT", "DELLA"))
  ) %>%
  select(-is_wt)
data
```

```
## # A tibble: 246 x 6
##       gid seed_count diameter height   weight genotype
##    <fctr>      <int>    <dbl>  <dbl>    <dbl>   <fctr>
##  1   WT-1        218    61.24  48.46  96.6179       WT
##  2   WT-1        152    65.91  54.16 123.3260       WT
##  3   WT-1        137    55.87  46.09  73.1770       WT
##  4   WT-1        106    60.80  50.83 100.0290       WT
##  5   WT-1        198    60.14  44.99  73.9210       WT
##  6   WT-1         65    57.75  46.99  81.8800       WT
##  7   WT-1        104    62.92  52.09 106.8040       WT
##  8   WT-1        112    54.27  44.16  68.6670       WT
##  9   WT-1        127    63.45  53.30 110.3500       WT
## 10   WT-1        119    36.97  47.80  43.6000       WT
## # ... with 236 more rows
```

## Check completeness of data

```
summary(data)
```

```
##           gid        seed_count        diameter         height      
##  ProD/ProD-3: 18   Min.   :  0.00   Min.   :17.71   Min.   : 21.59  
##  ProD/ProD-6: 16   1st Qu.: 45.00   1st Qu.:46.52   1st Qu.: 38.48  
##  WT-2       : 14   Median : 73.00   Median :53.31   Median : 43.91  
##  WT-5       : 14   Mean   : 74.42   Mean   :52.44   Mean   : 45.66  
##  WT-8       : 14   3rd Qu.:101.75   3rd Qu.:59.91   3rd Qu.: 48.82  
##  ProD/ProD-5: 14   Max.   :218.00   Max.   :73.44   Max.   :643.08  
##  (Other)    :156                    NA's   :1       NA's   :1       
##      weight         genotype  
##  Min.   :  6.883   DELLA:123  
##  1st Qu.: 43.531   WT   :123  
##  Median : 66.023              
##  Mean   : 66.414              
##  3rd Qu.: 86.788              
##  Max.   :155.800              
##
```

Looks like the `weight` and `height` data are missing 1 value each.

```
data %>%
  filter( is.na(weight) | is.na(height) )
```

```
## # A tibble: 1 x 6
##      gid seed_count diameter height weight genotype
##   <fctr>      <int>    <dbl>  <dbl>  <dbl>   <fctr>
## 1   WT-8         34       NA     NA  83.75       WT
```

## Some exploratory plots

### Seed count by genotype

```
ggplot(data) +
  aes(x = genotype,y = seed_count) +
  geom_boxplot() + 
  geom_jitter(aes(colour = gid), position = position_dodge(width = 0.5) )
```

### Diameter by genotype

```
ggplot(data) +
  aes(x = genotype, y = diameter) +
  geom_boxplot() + 
  geom_jitter(aes(colour = gid), position = position_dodge(width = 0.5) )
```

```
## Warning: Removed 1 rows containing non-finite values (stat_boxplot).
```

```
## Warning: Removed 1 rows containing missing values (geom_point).
```

### Height by genotype

```
ggplot(data) +
  aes(x = genotype, y = height) +
  geom_boxplot() + 
  geom_jitter(aes(colour = gid), position = position_dodge(width = 0.5) )
```

```
## Warning: Removed 1 rows containing non-finite values (stat_boxplot).
```

```
## Warning: Removed 1 rows containing missing values (geom_point).
```

Looks like there’s a strange datapoint - a height of > 600.

```
data %>% 
  filter(height > 600)
```

```
## # A tibble: 1 x 6
##           gid seed_count diameter height weight genotype
##        <fctr>      <int>    <dbl>  <dbl>  <dbl>   <fctr>
## 1 ProD/ProD-9        108    52.48 643.08 61.802    DELLA
```

Let’s redo without that one point.

```
data %>%
  filter(height < 600) %>%
  ggplot() +
  aes(x = genotype, y = height) +
  geom_boxplot() + 
  geom_jitter(aes(colour = gid), position = position_dodge(width = 0.5) )
```

### Weight by genotype

```
ggplot(data) +
  aes(x = genotype, y = weight) +
  geom_boxplot() + 
  geom_jitter(aes(colour = gid), position = position_dodge(width = 0.5) )
```

## Linear model

First I’ll trim out the weird points we saw earlier

```
 trimmed_data <- data %>%
  filter( ! is.na(weight) | ! is.na(height) ) %>%
  filter(height < 600)
  
   model <- lm(seed_count ~ genotype + height + weight + diameter, data = trimmed_data)
   summary(model)
```

```
## 
## Call:
## lm(formula = seed_count ~ genotype + height + weight + diameter, 
##     data = trimmed_data)
## 
## Residuals:
##     Min      1Q  Median      3Q     Max 
## -63.744 -18.413  -3.864  17.801 108.358 
## 
## Coefficients:
##             Estimate Std. Error t value Pr(>|t|)    
## (Intercept)   1.5204    22.6549   0.067    0.947    
## genotypeWT   35.2993     3.8273   9.223  < 2e-16 ***
## height       -0.4884     0.4113  -1.187    0.236    
## weight        0.9201     0.2088   4.406 1.59e-05 ***
## diameter      0.2923     0.5922   0.494    0.622    
## ---
## Signif. codes:  0 '***' 0.001 '**' 0.01 '*' 0.05 '.' 0.1 ' ' 1
## 
## Residual standard error: 29.18 on 239 degrees of freedom
## Multiple R-squared:  0.5983, Adjusted R-squared:  0.5916 
## F-statistic: 88.98 on 4 and 239 DF,  p-value: < 2.2e-16
```

```
   plot(model)
```

## Summary

The WT samples have a significantly greater seed count than the DELLA, about 35 on average. The weight increases in WT by ~ 0.9 units (grams? probably). Diameter and height of the fruits are not affected.
