# Supplementary material for: Using CRISPR/Cas9 genome editing in tomato to create a gibberellin‐responsive dominant dwarf DELLA allele
Source: Plant Biotechnol J. 2018 Jun 22;17(1):132–40. doi: 10.1111/pbi.12952 (PMC6330640; doi:10.1111/pbi.12952)
Supplement: Supplementary file 4 — Data S1 Raw data, R Markdown and html documents. [file PBI-17-132-s001.zip › Data S1/Supporting data for Fig S3/flower_effects.html]

inflorescence\_effects.Rmd


# inflorescence\_effects.Rmd

#### *Dan MacLean*

#### *04/12/2017*

```
library(tidyverse)
library(stringr)
library(readxl)
```

```
data <- read_excel(
  "Inflorescence WT vs DELLA.xlsx", 
  skip = 1,
  col_types = c("numeric", "text", "numeric", "text", "numeric", "numeric", "numeric"),
  col_names = c("days", "plant",    "open_flowers", "genotype_char",    "flowers_set",  "truss",    "replicate")
) %>%
   mutate(
    plant = as.factor(plant),
    truss = as.factor(LETTERS[truss]),
    replicate = as.factor(letters[replicate]), 
    is_wt = str_detect( genotype_char, "WT"),
    genotype = factor(if_else(is_wt, "WT", "PROD"))
  ) %>%
  select(-is_wt, -genotype_char)
```

## Completeness of data

```
summary(data)
```

```
##       days            plant       open_flowers    flowers_set     truss   
##  Min.   : 56.00   1      : 344   Min.   : 0.00   Min.   : 0.000   A:1591  
##  1st Qu.: 72.00   2      : 344   1st Qu.: 0.00   1st Qu.: 0.000   B:1638  
##  Median : 79.00   3      : 344   Median : 2.00   Median : 5.000           
##  Mean   : 81.08   4      : 344   Mean   : 2.74   Mean   : 4.771           
##  3rd Qu.: 91.00   5      : 344   3rd Qu.: 5.00   3rd Qu.: 8.000           
##  Max.   :110.00   6      : 344   Max.   :19.00   Max.   :79.000           
##                   (Other):1165                                            
##  replicate genotype   
##  a:1045    PROD:1587  
##  b: 980    WT  :1642  
##  c: 624               
##  d: 580               
##                       
##                       
##
```

```
sum(! complete.cases(data))
```

```
## [1] 0
```

An unbalanced data set, more from `truss` `B` than `A`. Different numbers of measurements in `replicate`s `a - d` and different numbers of each `genotype`. All rows are complete.

### Open flowers over time

```
data %>%
ggplot() + aes(x = days, y = open_flowers) + geom_point( aes(colour = genotype)) + geom_smooth( aes(colour = genotype), se=F) + facet_wrap(~ genotype)
```

```
## `geom_smooth()` using method = 'gam'
```

Naively, the number of open flowers doesn’t look too much like it changes over genotype. However we have a strange set of peaks in those summary curves. Let’s look at it by plant / truss / replicate

```
data %>%
ggplot() + aes(x = days, y = open_flowers) + geom_point( aes(colour = plant)) + geom_smooth( aes(colour = plant), se=F) + facet_grid(truss ~ replicate )
```

```
## `geom_smooth()` using method = 'loess'
```

This is clearer, looks like there is an offset to the data, the days after first flower is the true start. Let’s calculate that per plant.

Note that `replicate` `c`, `truss` `B`, `plant` `3` has much higher points than the others.

## Correcting time offset

```
data <- data %>%
  group_by(truss, replicate, plant, genotype) %>%
  summarize( first_flower_day = min(days) ) %>%
  right_join(data, by.x = c("truss", "replicate", "plant","genotype"), by.y = c("truss", "replicate", "plant", "genotype")) %>%
  mutate(days_since_first  = days - first_flower_day )
```

```
## Joining, by = c("truss", "replicate", "plant", "genotype")
```

```
    ggplot(data) + aes(x = days_since_first, y = open_flowers) + geom_point( aes(colour = plant)) + geom_smooth( aes(colour = plant), se=F) + facet_grid(truss ~ replicate )
```

```
## `geom_smooth()` using method = 'loess'
```

Better, the peaks over time are more standard now. Now we can look at the genotype differences again.

## Open flowers over offset time

```
data %>%    
ggplot() + aes(x = days_since_first, y = open_flowers) + geom_point( aes(colour = genotype)) + geom_smooth( aes(colour = genotype), se=F) + facet_wrap(~ genotype)
```

```
## `geom_smooth()` using method = 'gam'
```

We’ve lost that double peak, and each curve looks more like we’d expect intuitively, with a single peak of opening, then fading over time. But there’s no clear effect of genotype. On aggregate, those `PROD` values look a little higher, but they’re suspiciously like the `replicate c truss B plant 3` numbers. Let’s make a graph that highlights that.

```
data %>%
  ggplot() + aes(days_since_first, open_flowers) + geom_point(aes(colour = plant, shape = replicate, size = truss)) + facet_wrap(~ genotype)
```

```
## Warning: Using size for a discrete variable is not advised.
```

Yup, this messy plot simply shows that those high points in the `PROD` are all from one plant in one replicate. That tricky `replicate c truss b plant 3` ### #is time to first flower different?

## Models

The appropriate model for `open_flowers ~ days_since_first`, looks like it might be non-linear, with a square term for `days_since_first`. We’ll try alternative model forms, a standard linear, a linear with a square term and a gam.

```
lm_fit <- lm(open_flowers ~  days_since_first + truss + replicate + plant + genotype, data = data)

lm_sq_fit <- lm(open_flowers ~  I(days_since_first ^ 2) + truss + replicate + plant + genotype, data = data)

library(gam)
```

```
## Loading required package: splines
```

```
## Loading required package: foreach
```

```
## 
## Attaching package: 'foreach'
```

```
## The following objects are masked from 'package:purrr':
## 
##     accumulate, when
```

```
## Loaded gam 1.14-4
```

```
gam_fit <- gam(open_flowers ~  s(days_since_first,3) + truss + replicate + plant + genotype, data = data)

anova(lm_fit, lm_sq_fit, gam_fit )
```

```
## Analysis of Variance Table
## 
## Model 1: open_flowers ~ days_since_first + truss + replicate + plant + 
##     genotype
## Model 2: open_flowers ~ I(days_since_first^2) + truss + replicate + plant + 
##     genotype
## Model 3: open_flowers ~ s(days_since_first, 3) + truss + replicate + plant + 
##     genotype
##   Res.Df   RSS     Df Sum of Sq     F    Pr(>F)    
## 1   3213 21602                                     
## 2   3213 20394 0.0000    1208.6                    
## 3   3211 16581 2.0001    3812.2 369.1 < 2.2e-16 ***
## ---
## Signif. codes:  0 '***' 0.001 '**' 0.01 '*' 0.05 '.' 0.1 ' ' 1
```

Looks like the best model is the GAM.

## GAM for open flowers

```
anova(gam_fit)
```

```
## Anova for Nonparametric Effects
##                        Npar Df Npar F     Pr(F)    
## (Intercept)                                        
## s(days_since_first, 3)       2 486.12 < 2.2e-16 ***
## truss                                              
## replicate                                          
## plant                                              
## genotype                                           
## ---
## Signif. codes:  0 '***' 0.001 '**' 0.01 '*' 0.05 '.' 0.1 ' ' 1
```

And this shows that there’s no evidence of effect of anything but `days_after_first`, including `genotype` on the number of open flowers over time. The only variable affecting flower count is time. Note that as this is all in `days_since_first`, there is no effect of the variables under test on the progression of flowering once started. It does not answer whether the days to first flowering are different, especially not by genotpye. We can answer that explicitly.

## Is earliest flowering affected by genotype?

```
data %>% 
  ggplot() + aes(genotype, first_flower_day) + geom_point(aes(colour = plant), position = position_dodge(width = 0.5 )) + facet_grid(replicate ~ truss)
```

Short answer, it appears not. But this is a little suspect. Why do all plants in reps/ trusses have the same first day of flowering?

## Flowers set over offset time

Let’s perform a similar analysis for the `flowers_set` variable. Which I take to be the number of flowers that set fruit. Again this will be modelled as a function of `days_since_first`.

```
data %>%
ggplot() + aes(x = days_since_first, y = flowers_set) + 
  geom_jitter( aes(colour = genotype))
```

Hmm, very high number of `flowers_set` data point somewhere!

```
data %>%
  filter( flowers_set > 60) %>%
  select(truss, replicate, plant, genotype, flowers_set)
```

```
## # A tibble: 1 x 5
## # Groups:   truss, replicate, plant [1]
##    truss replicate  plant genotype flowers_set
##   <fctr>    <fctr> <fctr>   <fctr>       <dbl>
## 1      A         d      1     PROD          79
```

So it’s `truss A replicate d plant 1 for PROD`. I imagine this is a typo, so I’ll screen it out.

```
data %>%
  filter( flowers_set < 70) %>%
ggplot() + aes(x = days_since_first, y = flowers_set) + 
  geom_jitter( aes(colour = genotype))
```

Let’s check all those high points in `PROD` aren’t coming from the same plant again.

```
data %>%
  filter( flowers_set < 70) %>%
  ggplot() + aes(days_since_first, flowers_set) + geom_point(aes(colour = plant, shape = replicate, size = truss)) + facet_wrap(~ genotype)
```

```
## Warning: Using size for a discrete variable is not advised.
```

Yep, those higher points in `PROD` are coming from the same ‘overactive’ plant as before. It could be worth looking into why this plant flowers so much more and sets more fruit!

## Models

Same approach as last time.

```
lm_fit2 <- lm(flowers_set ~  days_since_first + truss + replicate + plant + genotype, data = data)

lm_sq_fit2 <- lm(flowers_set ~  I(days_since_first ^ 2) + truss + replicate + plant + genotype, data = data)

library(gam)
gam_fit2 <- gam(flowers_set ~  s(days_since_first,3) + truss + replicate + plant + genotype, data = data)

anova(lm_fit2, lm_sq_fit2, gam_fit2 )
```

```
## Analysis of Variance Table
## 
## Model 1: flowers_set ~ days_since_first + truss + replicate + plant + 
##     genotype
## Model 2: flowers_set ~ I(days_since_first^2) + truss + replicate + plant + 
##     genotype
## Model 3: flowers_set ~ s(days_since_first, 3) + truss + replicate + plant + 
##     genotype
##   Res.Df   RSS     Df Sum of Sq      F    Pr(>F)    
## 1   3213 18189                                      
## 2   3213 24509 0.0000   -6320.4                     
## 3   3211 16411 2.0001    8098.7 792.28 < 2.2e-16 ***
## ---
## Signif. codes:  0 '***' 0.001 '**' 0.01 '*' 0.05 '.' 0.1 ' ' 1
```

Looks like the best model is the GAM.

## GAM for open flowers

```
anova(gam_fit2)
```

```
## Anova for Nonparametric Effects
##                        Npar Df Npar F     Pr(F)    
## (Intercept)                                        
## s(days_since_first, 3)       2 173.97 < 2.2e-16 ***
## truss                                              
## replicate                                          
## plant                                              
## genotype                                           
## ---
## Signif. codes:  0 '***' 0.001 '**' 0.01 '*' 0.05 '.' 0.1 ' ' 1
```

And again, no effect of anything but time on fruits.

## Final plots

```
library(ggthemes)
data %>%    
ggplot() + aes(x = days_since_first, y = open_flowers) + geom_jitter( aes(colour = genotype), alpha=0.4) + geom_smooth( aes(colour = genotype), method = "loess", weight = 4 ) + theme_bw() + scale_colour_ptol()
```

```
data %>%    
ggplot() + aes(x = days_since_first, y = flowers_set) + geom_jitter( aes(colour = genotype), alpha=0.4) + geom_smooth( aes(colour = genotype), method = "loess", weight = 4 ) + theme_bw() + scale_colour_ptol()
```
